# Enhanced photovoltaic property by forming p-i-n structures containing Si quantum dots/SiC multilayers

**DOI:** 10.1186/1556-276X-9-634

**Published:** 2014-11-25

**Authors:** Yunqing Cao, Peng Lu, Xiaowei Zhang, Jun Xu, Ling Xu, Kunji Chen

**Affiliations:** 1 National Laboratory of Solid State Microstructures and School of Electronic Science and Collaborative Innovation Center of Advanced Microstructures, Nanjing University, Nanjing 210093, China

**Keywords:** Si quantum dots (Si QDs), Silicon carbide, Multilayers, Solar cell

## Abstract

Si quantum dots (Si QDs)/SiC multilayers were fabricated by annealing hydrogenated amorphous Si/SiC multilayers prepared in a plasma-enhanced chemical vapor deposition system. The thickness of amorphous Si layer was designed to be 4 nm, and the thickness of amorphous SiC layer was kept at 2 nm. Transmission electron microscopy observation revealed the formation of Si QDs after 900°C annealing. The optical properties of the Si QDs/SiC multilayers were studied, and the optical band gap deduced from the optical absorption coefficient result is 1.48 eV. Moreover, the p-i-n structure with n-a-Si/i-(Si QDs/SiC multilayers)/p-Si was fabricated, and the carrier transportation mechanism was investigated. The p-i-n structure was used in a solar cell device. The cell had the open circuit voltage of 532 mV and the power conversion efficiency (PCE) of 6.28%.

**PACS:**

81.07.Ta; 78.67.Pt; 88.40.jj

## Background

Recently, Si-based solar cells have been widely used due to its abundance, contaminant-free, and mature fabrication process. However, for a single p-n junction crystalline silicon solar cell, the maximum theoretical power conversion efficiency is only 29.8%, because of the inevitable longer and shorter wavelength loss [1]. Since the low-dimensional Si quantum dots (Si QDs) have quantum confinement effect, the band gap of Si QDs can be tunable by controlling the dot size, which provides an effective way to adjust the energy band structures by changing the Si QDs size to get better spectral matching [2,3]. It was reported that the broadband spectral absorption can be realized by forming the all Si-based tandem type solar cells, whose power conversion efficiency can exceed the Shockley-Queisser limit [4-6]. In the present stage, it is interesting to study the photovoltaic properties of Si QDs-based structures for their actual applications in devices. For example, Park et al. studied Si QDs/c-Si heterojunction solar cells based on Si-rich silica (SRO)/SiO_2_ multilayers and observed that the shift of the spectral response range with changing the size of Si QDs from 3 to 8 nm [7]. However, the large band offset between Si and SiO_2_ causes the low carrier tunneling probability, which may deteriorate the performance of devices.

Compared with SiO_2_, amorphous silicon carbide (a-SiC) has a lower band gap, which is helpful for enhancing the carrier transport efficiency to improve the device performance [8]. Recently, the structural and physical properties of Si QDs embedded in amorphous SiC matrix have been studied [9,10]. Kurokawa et al. found that Si QDs can be formed by thermal annealing amorphous SiC films, and the photoluminescence band was redshifted with increasing Si QDs size [11]. Song et al. designed the p-type Si QDs: SiC/n-type c-Si heterojunction device and achieved the open circuit voltage, short circuit current density, and fill factor of 463 mV, 19 mA/cm^2^, and 53%, respectively [12].

In our previous works, Si QDs/SiO_2_, Si QDs/SiN_x_, and Si QDs/SiC multilayers were fabricated by thermal annealing of amorphous Si/SiO_2_, Si/SiN_x_, and Si/SiC multilayered structures [13-15]. An intense electroluminescence (EL) was achieved in Si QDs/SiC multilayers and the EL peak energy redshifted with increasing the Si QDs size, which indicated the quantum confinement effect [16,17]. In the present work, hydrogenated amorphous Si (a-Si:H)/SiC multilayers were fabricated in a conventional plasma-enhanced chemical vapor deposition (PECVD) system. The thickness of a-Si:H was designed to be 4 nm, and the thickness of amorphous SiC layer was 2 nm. Due to the constrained crystallization principle, Si QDs with controllable size were achieved by annealing the as-deposited samples at 900°C for 1 h. The microstructures of samples before and after annealing were examined by cross-sectional transmission electron microscopy (x-TEM) and Raman spectroscopy, which revealed the formation of Si QDs after thermal annealing. The optical properties were characterized by optical absorption measurements. Moreover, the p-i-n structure with n-a-Si/i-(Si QDs/SiC multilayers)/p-Si was fabricated, which exhibits the photovoltaic properties with the power conversion efficiency of 6.28%.

## Methods

The a-Si:H/SiC multilayers (MLs) with six periods were fabricated on quartz and p-Si substrates in a PECVD system. The a-Si sublayer was deposited by using pure silane (SiH_4_), while the a-SiC layer was deposited by using a gas mixture of SiH_4_ and methane (CH_4_) with the gas ratio *R* (*R* = [CH_4_]/[SiH_4_]) of 10. In our previous work, it was found that the collection efficiency of photo-generated carriers was improved by reducing the thickness of SiC barriers [18]. In the present case, the thickness of a-Si:H layer was designed to be 4 nm, and the thickness of amorphous SiC layer was kept at 2 nm. During the deposition process, the radio frequency power and the substrate temperature was kept at 30 W and 250°C, respectively. The post-treatment performed in N_2_ atmosphere included two steps: dehydrogenation at 450°C for 1 h and subsequently annealing at 900°C for 1 h. The structural change of the Si/SiC MLs before and after annealing was evaluated by Raman spectroscopy (Jobin Yvon Horiba HR800 spectrometer, Kyoto, Japan). The formation of Si QDs was determined by transmission electron microscopy (TEM) using Technai G2 operated at 200 KV. The optical absorption of the Si/SiC MLs was measured at room temperature by Shimadzu UV-3600 spectrophotometer (Shimadzu UV-3600 spectrophotometer, Kyoto, Japan).

All Si-based solar cell containing Si QDs (4 nm)/SiC (2 nm) MLs and 10 nm-thick phosphorus-doped amorphous Si layer were fabricated on p-type Si wafer (with a thickness of 450 μm and a resistivity in the range of 1 to 3 Ω•cm) to get p-i-n solar cell structure. The n-layer was deposited by a gas mixture of phosphorane (PH_3_) (1% in H_2_) and SiH_4_ with the ratio of [PH_3_]/[SiH_4_] = 10. As a reference, a-Si/SiC multilayer-based p-i-n structure was also fabricated at the same time. Al electrode was evaporated on both the surface and rear side of p-type Si wafer. The cell area was about 0.8 cm^2^. The illuminated current-voltage (I*-*V) characteristics of the cell device were measured under an AM 1.5 (100 mW/cm^2^) illumination by using a Keithley 610C electrometer (Keithley 610C electrometer, Minato-ku, Japan).

## Results and discussion

**Figure 1:**
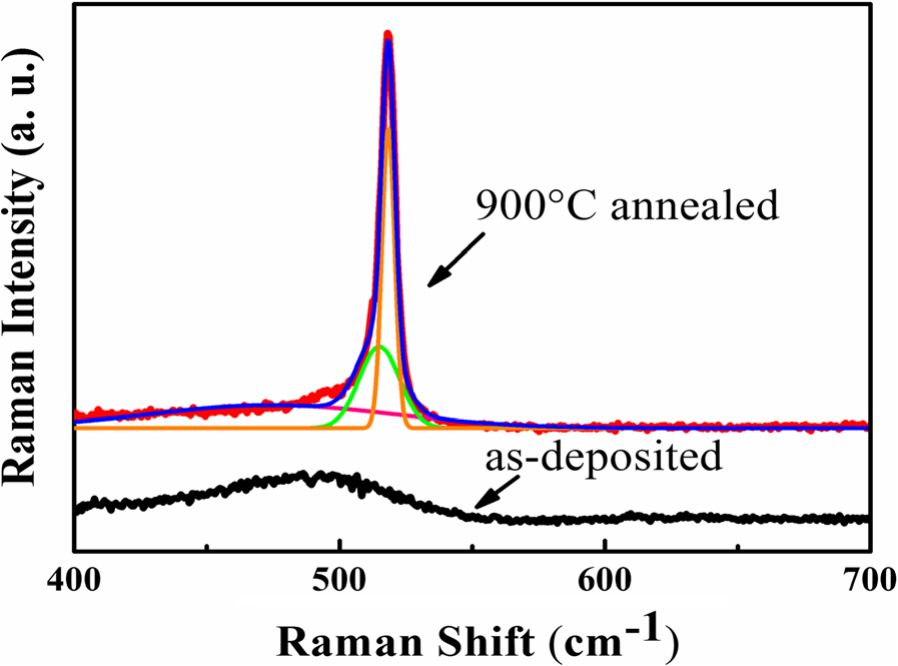
**Raman spectra of samples.** As-deposited Si/SiC multilayers (black line) and 900°C annealed Si/SiC multilayers (red line).

**Figure 2:**
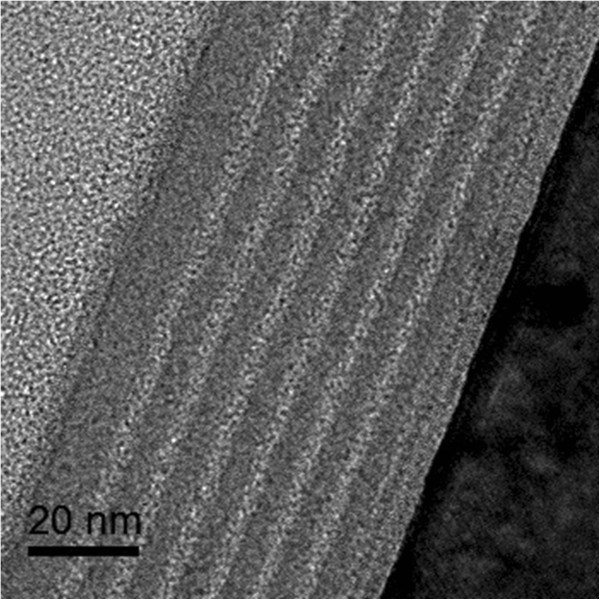
Cross-sectional TEM image of as-deposited a-Si (4 nm)/a-SiC (2 nm) multilayers.

**Figure 3:**
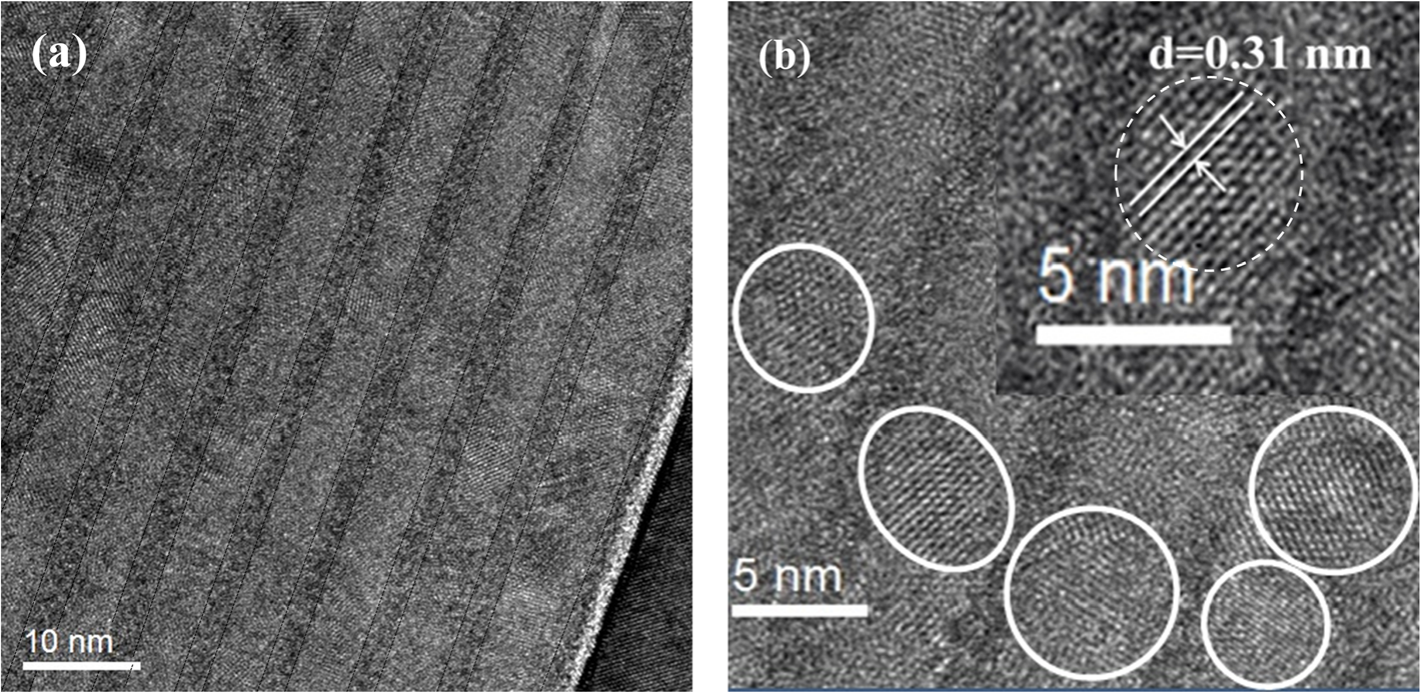
**Cross-sectional TEM image of 900°C annealed Si (4 nm)/SiC (2 nm) multilayers. (a)** The cross-sectional TEM image of Si QDs/SiC MLs after 900 °C annealing. **(b)** The high-resolution TEM image, in which the formed Si QDs can be clearly identified.

Figure 1 shows the Raman spectra of as-deposited and 900°C annealed multilayered samples. It is noted that only one broad band centered at 480 cm^-1^ exists in the as-deposited sample, which is attributed to the transverse optical (TO) mode of amorphous Si-Si bonds. However, an intense peak at 517 cm^-1^ associated with crystallized Si TO mode appears for 900°C annealed sample, which indicates that the amorphous Si layers have been crystallized to form nano-crystallized Si. In order to estimate the crystallinity ratio and size of Si QDs, we fitted the Raman spectrum via the Gaussian deconvolution by three components, which is located at 480, 510, and 520 cm^-1^. The crystallinity ratio (Xc) is figured out as 49.5% by integrated Gaussian peaks of 520 and 480 cm^-1^[19]. The average size of Si crystals is about 4.8 nm, according to the phonon confinement model [20], which indicates the formation of nano-crystalline Si quantum dots.The cross-sectional TEM measurements were performed to further characterize the multilayered structures before and after annealing. Figure 2 is the cross-sectional TEM image of as-deposited a-Si (4 nm)/a-SiC (2 nm) MLs. The layered structures and smooth interfaces of Si/SiC can be clearly identified. The thickness of a-SiC sublayer is 1.9 nm and the thickness of a-Si sublayer is 4.2 nm, respectively, which is very close to the pre-designed value estimated from the deposition rate. Figure 3a shows the cross-sectional TEM image of Si QDs/SiC MLs after 900°C annealing. The periodically layered structures are well kept and the interfaces are still smooth and abrupt. The total thickness of the Si QDs/SiC MLs is about 40 nm. The formation of Si QDs in a-Si layers can be identified in the high-resolution TEM image. As given in Figure 3b, the average size is about 5 nm, which is well agreement with the Raman result. As indicated in the inset of Figure 3b, the crystalline interplanar spacing is 0.31 nm of formed Si QDs, which suggests the Si (111) crystalline faces.

**Figure 4:**
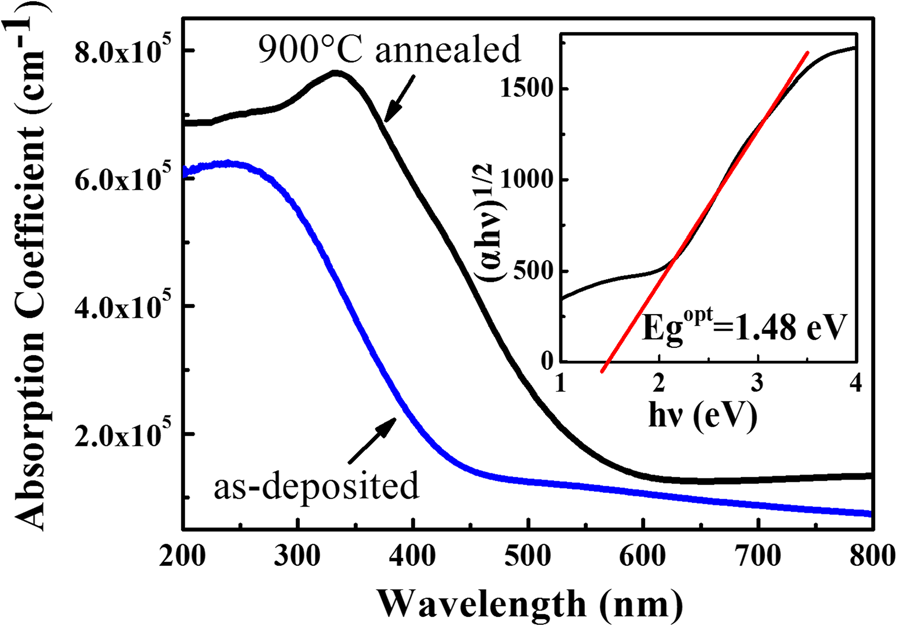
**Optical absorption coefficient spectra of Si/SiC multilayers.** As-deposited (blue line) and 900 °C annealed samples (black line). The inset is the (αhν)^1/2^ ~ hν relationship of 900°C annealed sample.

The optical properties of Si/SiC MLs deposited on quartz substrate before and after annealing are studied by measuring the optical transmission spectra and reflection spectra in the spectral range of 200 to 800 nm. The optical absorption coefficient α is calculated and given in Figure 4. It is found that the optical absorption of as-deposited a-Si/SiC MLs is quite high, which is above 10^5^ cm^-1^ when the wavelength is less than 400 nm. However, the absorption coefficient of 900°C annealed Si QDs/SiC MLs is much higher in the whole visible light region (300 to 800 nm), which indicates that the Si QDs/SiC MLs can strongly absorb the visible light photons, especially in short-wavelength range. Based on the Tauc model, the optical band gap of Si QDs/SiC MLs can be deduced from the linear fitting of (αhν)^1/2^ ~ hν relationship [21], as shown in the inset of Figure 4. The deduced optical band gap of our MLs after 900°C annealing is 1.48 eV, which is blueshifted compared to that of crystallized Si, which can be attributed to the quantum size effect [22]. In our previous work, we found that the optical band gap of Si QDs/SiC MLs is enlarged with reducing the dot size, which resulted in the blueshift of electroluminescence peaks. We used a modified effective mass approximation (EMA) model to estimate the band gap of Si QDs/SiC MLs instead of infinite barrier model by considering the Coulomb effect and the correlation energy terms [23]. Based on this model, the optical band gap (*E*_g_^opt^) can be expressed as follows:

$${E_{g}}^{\text{opt}}=1.12\left(\mathrm{eV}\right)+\Delta E_{e,h,\text{reduced}}-\frac{0.4512}{R}-0.003394\left(\mathrm{eV}\right)$$

where 1.12 is the band gap of crystalline silicon, *R* is the average diameter of Si QDs, 0.4512 is the Coulomb coefficient, 0.003394 is the correlation energy terms, and ΔE_e,h,reduced_ is the decreased confinement energy related with the barrier height in conduction and valence band. According to the formula, the estimated band gap of Si QDs with dot size of 4 to 5 nm is 1.4 to 1.5 eV, which is in agreement with the experimental result.

**Figure 5:**
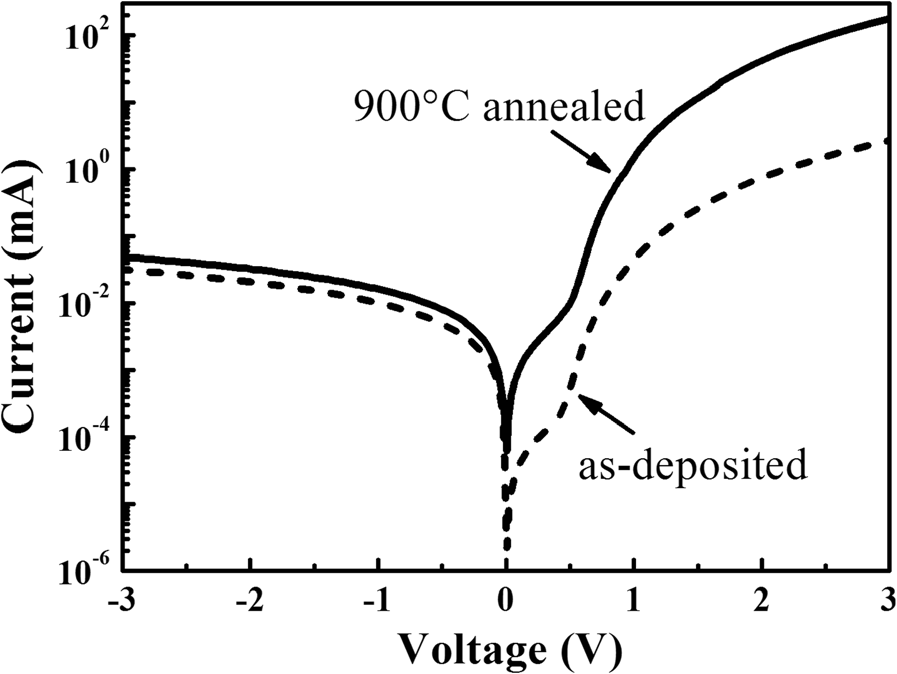
**Current-voltage relationships of p-i-n structures.** As-deposited (dashed line) and 900°C annealed samples (solid line) with p-i-n structures.

Based on the structural and optical properties of prepared Si/SiC MLs, we fabricated the p-i-n device structures containing phosphorus-doped a-Si: H and Si QDs/SiC (or a-Si/SiC) MLs on p-Si substrates. Figure 5 shows the current-voltage (I-V) relationships of p-i-n structures with and without annealing. The rectification characteristics are clearly observed for both samples which indicate that the p-i-n structures are well formed by the present approach. Compared with that of as-deposited sample, the reverse current of annealed sample is in the same order (approximately 10^-2^ mA at -3 V) while the forward current is increased by almost two orders of magnitude (from 10^0^ to 10^2^ mA at +3 V), which indicates that the rectification ratio of 900°C annealed p-i-n structure reaches to 2 × 10^3^ at the applied voltage *V* = ±3 V. The increase in the forward current of annealed p-i-n sample can be attributed to the increase of the conductivity in crystallized Si layers. In our previous work, we found that the dark conductivity of prepared a-Si: H film is about 4 × 10^-9^ S/cm and the conductivity reaches to 2 × 10^-7^ S/cm after 1,000°C thermal annealing [24]. It is reasonable to assume that the resistance in Si QDs/SiC MLs is also reduced after annealing due to the enhancement in the carrier transport properties, which results in the improvement of the rectification ratio of annealed p-i-n structure containing Si QDs/SiC MLs.

**Figure 6:**
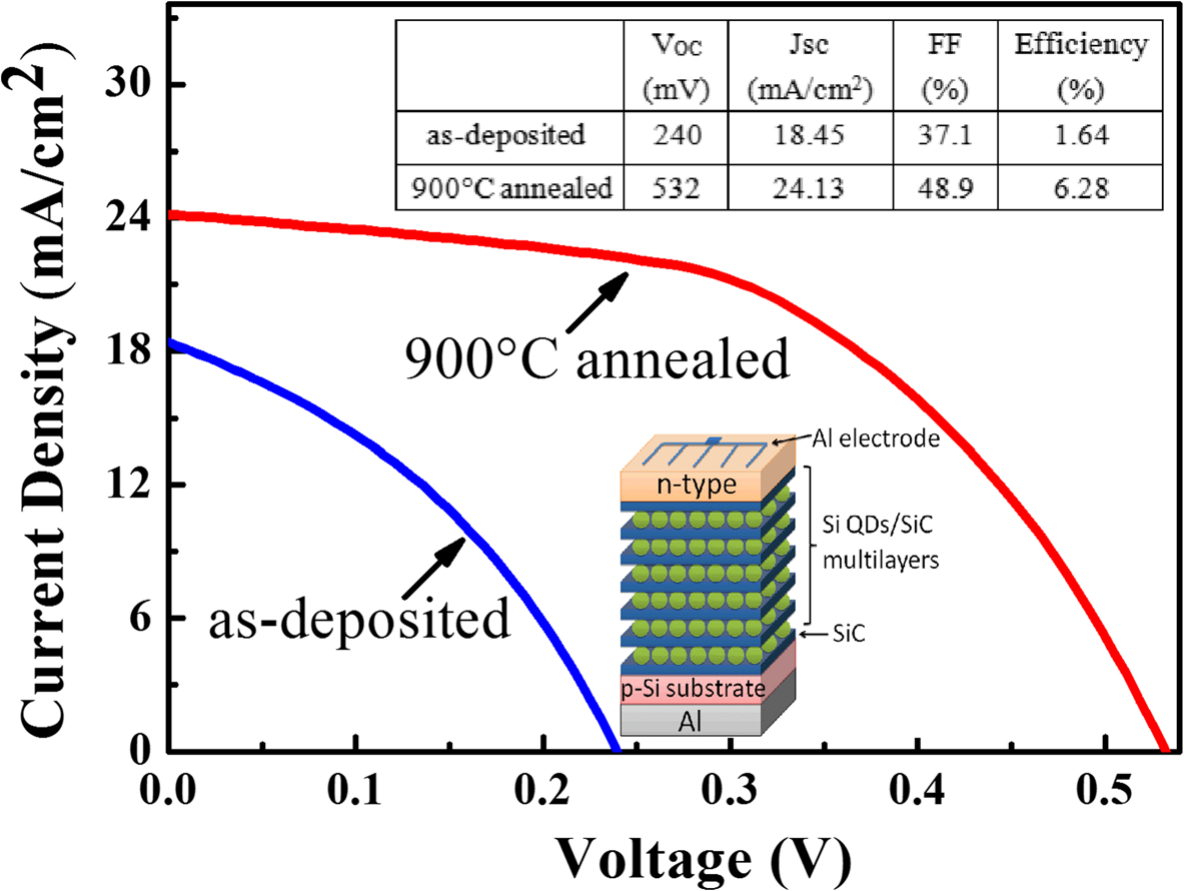
**One-sun-illuminated current-voltage curves of solar cells.** As-deposited (blue line) and 900°C annealed samples (red line) with p-i-n structures; inset is the schematic diagram of the device structure.

The prototype solar cell devices were fabricated by evaporating the Al strip-shaped electrode on the p-i-n structures, and the cell area is about 0.8 cm^2^. Figure 6 is the AM 1.5 (100 mW/cm^2^) illuminated I-V curves of solar cells based on as-deposited and 900°C annealed p-i-n structures, and inset of Figure 6 is the schematic diagram of the device structures. As shown in Figure 6, the open circuit voltage (*V*_oc_), short circuit current density (*J*_sc_), and fill factor (FF) for the device based on as-deposited p-i-n structure is 240 mV, 18.45 mA/cm^2^, and 37.1%, respectively. The power conversion efficiency (PCE) is about 1.64%. However, the device performance of p-i-n structure containing Si QDs/SiC MLs after annealing is significantly enhanced. The *V*_oc_ is increased to 532 mV, and the *J*_sc_ is increased to 24.13 mA/cm^2^ with the FF of 48.9%; the PCE reaches to 6.28%. The increased *V*_oc_ of the annealed cell compared to that of as-deposited one can be attributed to the increased film quality as well as the improved p-i-n structure after annealing. For as-deposited sample containing a-Si: H/SiC MLs, high density of defect states, such as dangling bonds and interface states between amorhous Si and SiC layers, may lead to the pining of the Fermi levels which results in the low *V*_oc_. After high-temperature annealing, the film quality can be significantly improved [25], which is helpful for increasing *V*_oc_ by reducing the defect states. Moreover, post-annealing can also improve the p-i-n structure. As shown in Figure 5, the obvious rectification behavior can be observed in the annealed p-i-n structure. The shunt resistance of cell device is increased from 56 Ω to 108 Ω after annealing, which also indicates the improved p-i-n structure after annealing, due to the improved interface quality and the enhanced doping effect in n-a-Si layer by thermally activating dopants. The present result is obviously improved compared with that of solar cell based on Si QDs/SiO_2_ MLs [26] and is compatible with the reported values with other groups by using Si QDs/SiC. For example, Song et al. designed the p-type Si QDs: SiC/n-type c-Si heterojunction device and achieved the *V*_oc_, *J*_sc_, and PCE of 463 mV, 19 mA/cm^2^, and 4.66%, respectively [12]. The improvement in the cell performance for annealed sample can be attributed to the formation of Si QDs after 900°C annealing, which enhances the photon-generated carrier separation and carrier transportation properties due to the improved electronic property of p-i-n structure.

**Figure 7:**
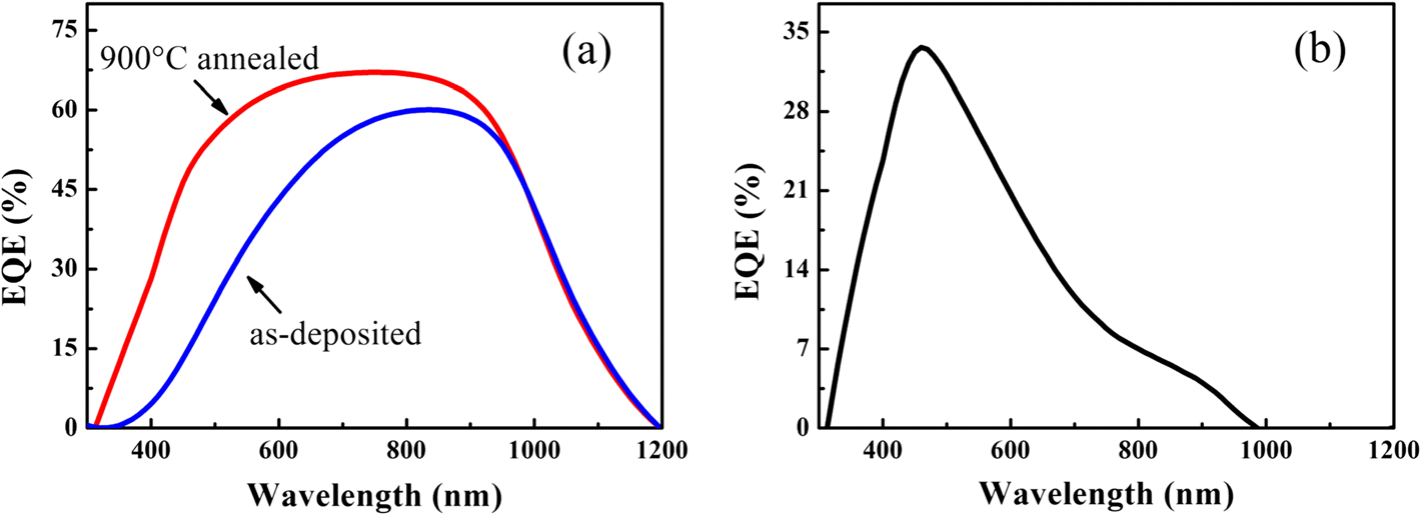
**External quantum efficiency of solar cells. (a)** The EQE results of p-i-n device structures containing as-deposited (blue line) and 900°C annealed (red line) Si/SiC MLs. **(b)** The EQE results of p-i-n solar cell containing Si QDs/SiC MLs by subtracting the EQE of cell containing as-deposited one.

In order to further understand the role of Si QDs in the present cell device, we measured the external quantum efficiency (EQE) of p-i-n device sample and compared with that of the as-deposited one. Figure 7a is the EQE of p-i-n device structures containing as-deposited and 900°C annealed Si/SiC MLs in the spectral range of 300 to 1,200 nm. It is found that in the long wavelength region, the EQE results for two cells are almost the same. However, the EQE is significantly improved in the whole visible light region (300 to 900 nm), which can be attributed to the contribution of the formed Si QDs in annealed sample. In our cell devices, part of the carriers was generated from Si substrates with the incident photons with long wavelength. They contributed to the *J*_sc_ both in as-deposited and annealed cells. In order to further investigate the contribution of Si QDs, we give the EQE result of p-i-n solar cell containing Si QDs/SiC MLs by subtracting the EQE of cell containing as-deposited one. As shown in Figure 7b, the improved EQE is located at the spectral range of 300 to 1,000 nm with the peak at 500 nm. As mentioned before, the optical band gap of the Si QDs/SiC MLs in our case is about 1.5 eV (approximately 820 nm), and the incident photons with high energy can be effectively absorbed by Si QDs to generate the electron-hole pairs which are separated by the built-in field in p-i-n structure. The electrons and holes can be effectively collected in the annealed sample due to its improved carrier transportation properties as shown in Figure 5. It is worth noting that parameters of the present cells have not been optimized, and there is no any light trapping structures used. The optimized design and processing control can further improve the cell performance.

## Conclusions

In summary, we fabricated Si QDs/SiC MLs by annealing a-Si: H/SiC MLs at 900°C with amorphous Si thickness of 4 nm and amorphous SiC thickness of 2 nm. Cross-sectional TEM observation reveals that the Si QDs were formed after annealing, and the average dot size is around 4.8 nm. It is found that the optical absorption edge is blueshifted compared with the c-Si, and the optical band gap is about 1.48 eV, which is well agreement with the theoretical estimation by using modified EMA model. Moreover, the p-i-n device structures containing Si/SiC MLs were fabricated. Improved rectification characteristics were observed in annealed sample compared with that in as-deposited one and the rectification ratio is about 2,000. The enhanced photovoltaic properties were observed in both annealed and as-deposited p-i-n cell devices. The cell containing Si QDs/SiC MLs has the *V*_oc_ of 532 mV, *J*_sc_ of 24.1 mA/cm^2^, and PCE of 6.28%. The improvement in EQE result for annealed cell device can be attributed to the formation of Si QDs which enhances the absorption of incident photons, especially in the short wavelength range and the carrier transportation process. Our experiment results infer that the Si QDs/SiC MLs can be used as a potential candidate for advanced optoelectronic devices.

## Abbreviations

EQE: External quantum efficiency; PCE: Power conversion efficiency; Si QDs/SiC MLs: Si quantum dots/SiC multilayers.

## Competing interests

The authors declare that they have no competing interests.

## Authors’ contributions

YQC and JX conceived the idea and carried out the experiments. YQC and PL participated in the preparation of the samples. YQC, XWZ, and JX took part in the experiments and the discussion of the results. YQC drafted the manuscript with the instruction of JX, LX, and KJC. All authors read and approved the final manuscript.
